# Leptin Induces Apoptotic and Pyroptotic Cell Death via NLRP3 Inflammasome Activation in Rat Hepatocytes

**DOI:** 10.3390/ijms222212589

**Published:** 2021-11-22

**Authors:** Ananda Baral, Pil-Hoon Park

**Affiliations:** 1 College of Pharmacy, Yeungnam University, Gyeongsan 38541, Korea; ananbaral@gmail.com; 2 Research Institute of Cell Culture, Yeungnam University, Gyeongsan 38541, Korea

**Keywords:** autophagy, ER stress, hepatocyte, inflammasomes, leptin

## Abstract

Leptin, a hormone that is predominantly produced by adipose tissue, is closely associated with various liver diseases. However, there is a lack of understanding as to whether leptin directly induces cytotoxic effects in hepatocytes as well as the mechanisms that are involved. Inflammasomes, which are critical components in the innate immune system, have been recently shown to modulate cell death. In this study, we examined the effect of leptin on the viability of rat hepatocytes and the underlying mechanisms, with a particular focus on the role of inflammasomes activation. Leptin treatment induced cytotoxicity in rat hepatocytes, as determined by decreased cell viability, increased caspase-3 activity, and the enhanced release of lactate dehydrogenase. NLRP3 inflammasomes were activated by leptin both in vitro and in vivo, as determined by the maturation of interleukin-1β and caspase-1, and the increased expression of inflammasome components, including NLRP3 and ASC. Mechanistically, leptin-induced inflammasome activation is mediated via the axis of ROS production, ER stress, and autophagy. Notably, the inhibition of inflammasomes by treatment with the NLRP3 inhibitor or the IL-1 receptor antagonist protected the hepatocytes from leptin-induced cell death. Together, these results indicate that leptin exerts cytotoxic effects in hepatocytes, at least in part, via the activation of NLRP3 inflammasomes.

## 1. Introduction

Leptin, a hormone that is primarily derived from adipose tissue, possesses diverse and complex metabolic signaling potential, and there is a growing appreciation that elevated plasma leptin levels are related to numerous metabolic disorders [1]. In addition, a growing body of recent evidence has suggested that leptin signaling is also implicated in the development and progression of various types of liver diseases via multiple mechanisms. For example, leptin activates hepatic stellate cells, which leads to the up-regulation of collagen expression and promotes hepatic fibrosis [2]. It also stimulates Kupffer cells, resident macrophages in the liver, leading to inflammatory responses and the development of steatohepatitis [3]. Moreover, leptin modulates lipid metabolism in hepatocytes, resulting in hepatic steatosis [4]. Thus, it has been well documented that leptin induces various pathophysiological conditions in the liver by modulating several signaling cascades in different liver cells. However, it is unclear whether leptin directly causes cytotoxicity in hepatocytes, and the underlying mechanisms are not well defined.

Inflammasomes play an important role in the innate immune system. They are composed of a sensor protein (e.g., nod-like receptor), adaptor protein (apoptosis-associated speck-like protein containing a caspase-1 recruitment domain (ASC)), and caspase-1. Through the concerted actions of these components, inflammasomes act as a signaling platform for the production of inflammatory cytokines upon exposure to pathogenic stimuli, such as exogenous pathogen-associated molecular patterns (PAMPs) and endogenous damage-associated molecular patterns (DAMPs) [5]. The sensing of inflammatory signals initiates the assembly of inflammasome components, which causes the recruitment of caspase-1 and the maturation of the interleukin family of cytokines, including IL-1β, IL-18, and IL-33 [6]. Increasing recent evidence demonstrates that inflammasomes are present in parenchymal hepatocytes as well as in innate immune cells in the liver and that the production of inflammatory cytokines plays a role in various pathogenesis, including drug-induced liver injury [7], ischemia-reperfusion liver injury [8], and non-alcoholic fatty liver disease [9,10]. Inflammasome activation leads to the activation of the caspase family and causes a pro-inflammatory type of programmed cell death, known as pyroptosis and apoptosis [11]. Notably, in contrast to this notion, recent studies have demonstrated that inflammasome activation is implicated in the survival and proliferation of cells [12], most of which are observed in cancer models, suggesting that inflammasomes modulate cell death and survival in a complex manner and that their roles depend on the experimental conditions.

The endoplasmic reticulum (ER) is a central intracellular organelle that is responsible for protein folding, which is required for protein transport to the Golgi apparatus and for their further secretion into the plasma [13]. Disturbances in ER functioning, collectively referred to as ER stress, result in an accumulation of misfolded or unfolded proteins in the ER lumen and can trigger the unfolded protein response (UPR). The UPR acts as the adative process for the restoration of normal cellular functions. However, under prolonged and excessive ER stress conditions, the UPR induces apoptotic cell death via the up-regulation of the C/EBP homologous protein (CHOP), the down-regulation of Bcl-2, and the translocation of Bax from the mitochondria to the cytosol [14]. Previous studies have demonstrated that excessive ER stress is closely associated with hepatocytes death and leads to inflammasome activation through various mechanisms, including thioredoxin interactingt-dependent manner [15] and dynamin-related protein-mediated mitochondrial fission [16].

Autophagy is a highly conserved self-digestive process that is responsible for removing dysfunctional cellular components via the sequestration of the substrates into double-membrane vesicles (autophagosomes) followed by fusion with lysosomes [17]. Autophagy was observed in dying cells accompanied with the structural features of cell death, such as the loss of organelles and nuclear shedding, and therefore, it was originally reported as a distinct type of cell death [18]. However, increasing recent evidence also indicates that the breakdown of target proteins or intracellular organelles provides the nutrient sources and building blocks for the synthesis of new proteins [19], suggesting that autophagy is crucial for the maintenance of cellular homeostasis and acts as an adaptive process for cells that have been exposed to the stressful conditions [20], suggesting multi-functional roles of autophagy in the modulation of cell death and survival, which could be determined in a context-dependent manner.

Based on findings from previous studies, leptin signaling is implicated in various hepatic pathological conditions. To better understand the mechanisms underlying leptin-induced hepatic damage, we examined whether leptin exerts cytotoxic effects in hepatocytes and elucidated the underlying mechanisms. Herein, we observed that leptin directly induces apoptotic and pyroptotic cell death in rat hepatocytes in an inflammasome-dependent manner. In addition, leptin-induced inflammasome activation is mediated, at least in part, through the axis of ROS production, ER stress, and autophagy induction.

## 2. Results

### 2.1. Leptin Induces Cell Death in Rat Hepatocytes

**Figure 1:**
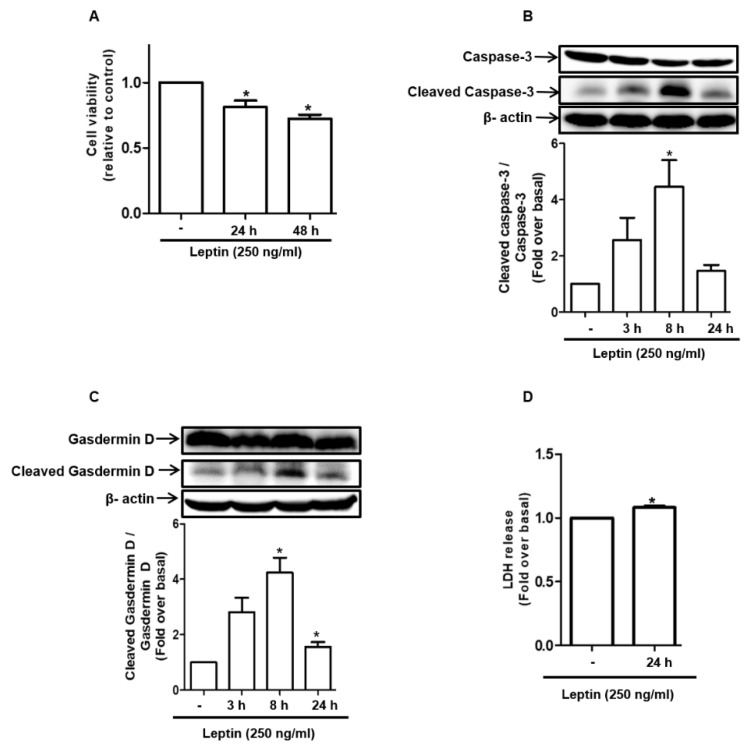
Cytotoxic effects of leptin in rat hepatocytes: Rat hepatocytes were isolated and treated with leptin (250 ng/mL) for the indicated time periods. (**A**) Cell viability was determined by MTS assay. (**B**) Cleaved and total caspase-3 levels were measured by Western blot analysis. Representative images from three independent experiments are shown along with β-actin as an internal control. Expression levels of active caspse-3 were quantitated by densitometric analysis and presented in the lower panel. (**C**) Total and cleaved gasdermin D expression levels were determined by Western blot analysis. Representative images from three independent experiments are shown. Expression levels of cleaved gasdermin D were quantified by densitometric analysis and are presented in the lower panel. (**D**) Cell culture media were collected and used to measure the levels of LDH released. Values are presented as fold changes compared to the control group and are expressed as mean ± SEM, *n* = 3. * denotes *p* < 0.05 compared with control cells.

To investigate whether leptin directly exerts cytotoxic effects in hepatocytes, we examined the effect of leptin on the viability of rat hepatocytes. In this study, the cells were treated with 250 ng/mL of leptin, a concentration that was determined through the analysis of previously published literature and from our preliminary dose-dependent experiments. As shown in Figure 1A, leptin significantly reduced cell viability, as determined by MTS assay. In addition, leptin induced the activation of caspase-3 (Figure 1B), a marker of apoptosis, which caused the cleavage of Gasdermin D (Figure 1C), which is an executioner of pyroptosis and causes increases in the release of lactate dehyrogenase (LDH) (Figure 1D), which are considered pyroptotic cell death markers. These results suggest that leptin treatment exhibits cytotoxic effects in hepatocytes by inducing both apoptosis and pyroptosis. 

### 2.2. Cytotoxic Effect of Leptin in Hepatocytes Is Mediated via Inflammasome Activation

**Figure 2:**
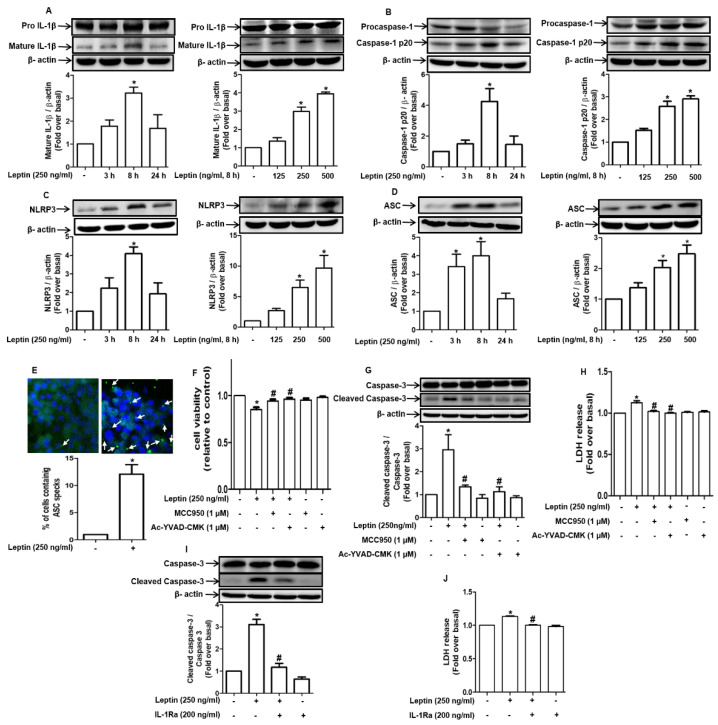
The critical role of inflammasome activation in cytototoxic effects of leptin in rat hepatocytes: (**A**–**D**) Hepatocytes isolated from rats were incubated with leptin (250 ng/mL) for the indicated time duration or different concentrations of leptin for 8 h. Expression levels of pro- and mature IL-1β (A), pro- and cleaved caspase-1 (**B**), NLRP3 (**C**), and ASC (**D**) expression levels were determined by Western blot. (**E**) Hepatocytes were treated with leptin (250 ng/mL) for 8 h in collagen-coated chamber slides. ASC speck formation was analyzed by fluorescent immunocytochemistry. Green dots indicated with white arrow and blue color represents ASC speck and DAPI, respectively. Representative images from three independent experiments are presented, and the levels of ASC speck formation were quantified, as shown in the lower panel. (**F**–**H**) Cells were pretreated with indicated concentrations of MCC950 or Ac-YVAD-CMK, a pharmacological inhibitor of NLRP3 and caspase-1, respectively, for 1 h followed by further incubation with leptin (250 ng/mL) for indicated time duration. (**F**) Cell viability was determined by MTS assay. (**G**) Expression levels of total and cleaved active caspase-3 were determined by Western blot. (**H**) Cell culture media were collected and used to measure the released LDH. (**I**,**J**) Rat hepatocytes were pretreated with IL-1 receptor antagonist (200 ng/mL) for 1 h followed by further stimulation with leptin for an additional 8 h. (**I**) Total and cleaved active caspase-3 levels were determined by Western blot. (**J**) The levels of LDH in the media were measured. For Western blot analyses, expression levels of the target genes were quantitated by densitometric analysis and are presented in the lower panel of each image. Values are presented as fold changes compared to the control group and are expressed as mean ± SEM, *n* = 3. In all of the the experiments, * denotes *p* < 0.05 compared to the control cells; # denotes *p* < 0.05 compared to the cells treated with leptin alone.

To elucidate the mechanisms by which leptin induces cytotoxicity in hepatocytes, we assessed the role of inflammasomes. For this, we first investigated if leptin induces the activation of inflammasomes and observed that leptin treatment markedly enhanced the levels of active mature IL-1β in a dose-dependent manner, showing a maximal effect at 8 h of treatment (Figure 2A, left and right panel, respectively). In addition, the levels of cleaved caspase-1 (p20) were also substantially increased in a time- and dose-dependent manner (Figure 2B, left and right panel). Moreover, leptin treatment prominently enhanced the expression of inflammasome subunits, including NLRP3 (Figure 2C) and ASC (Figure 2D). The polymerization of ASC into large helical fibril structures, which is referred to as ASC speck formation, is considered a marker for inflammasome activation [21]. As determined by immunocytochemical analysis, we further found that leptin significantly enhanced the ASC speck formation (Figure 2E). These results clearly indicate that leptin induces inflammasome activation in rat hepatocytes.

We next verified the functional role of inflammasome activation in leptin-induced hepatocyte death. As indicated in Figure 2F, pretreatment with pharmacological inhibitors of NLRP3 (MCC950) or caspase-1 (Ac-YVAD-CMK) restored the cell viability that had been decreased by leptin. Moreover, both leptin-induced caspase-3 activation and the increase in LDH release were abrogated through treatment with MCC950 and Ac-YVAD (Figure 2G,2H, respectively). Furthermore, pretreatment with an IL-1 receptor antagonist (IL-1 RA) blocked leptin-induced caspase-3 activation (Figure 2I) and LDH release (Figure 2J). These results collectively indicate that NLRP3 inflammasome activation mediates the cytotoxic effects of leptin in hepatocytes.

### 2.3. Inflammasome Activation by Leptin Is Mediated through ER Stress in Rat Hepatocytes 

**Figure 3:**
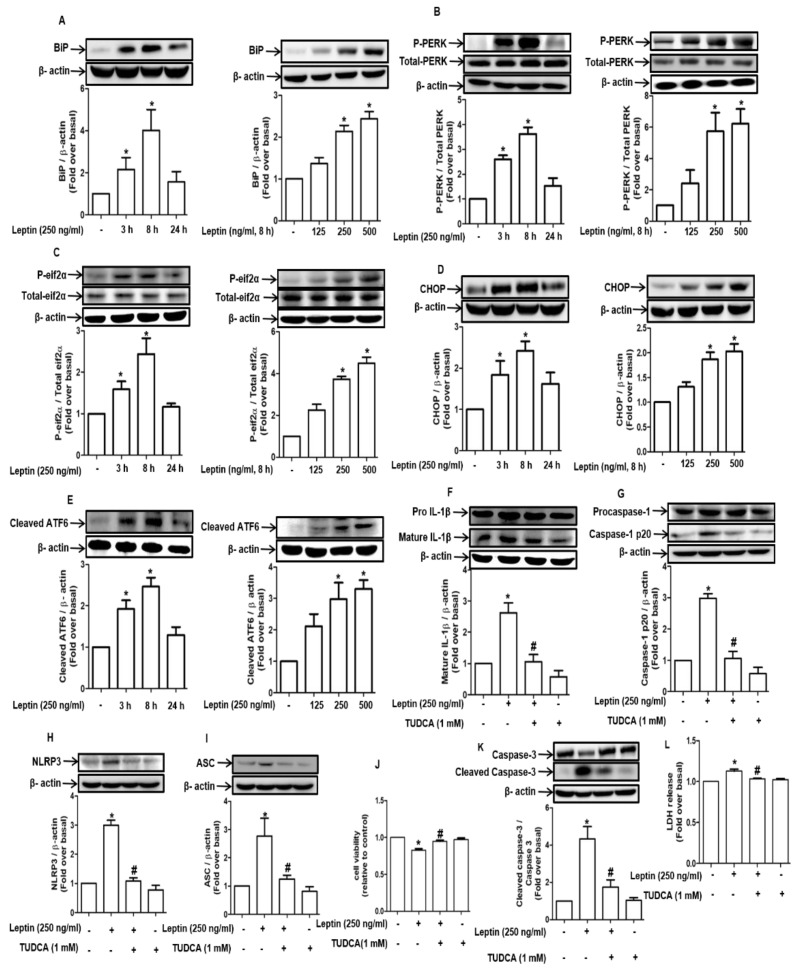
Involvement of ER stress in inflammasome activation by leptin in rat hepatocytes: (**A**–**E**) Rat hepatocytes were treated with leptin (250 ng/mL) for the indicated time duration or with different concentrations of leptin for 8 h. Dose- and time-dependent effects of leptin on the expression of various UPR proteins, including BiP (**A**), phospho PERK (**B**), phospho eif2α (**C**), CHOP (**D**), and cleaved ATF6 (**E**) were assessed by Western blot analysis. (**F**–**L**) Hepatocytes were pretreated with TUDCA (1 mM) for 1 h followed by further stimulation with leptin (250 ng/mL) for 8 h. Expression levels of IL-1β (**F**), caspase-1 (**G**), NLRP3 (**H**), and ASC (**I**) were measured by Western blot analysis. (**J**) Cell viability was determined by MTS assay. (**K**) Total and cleaved caspase-3 levels were measured by Western blot analysis. (**L**) The amount of LDH that was secreted was assessed. For Western blot analyses, expression levels of the genes were quantitated by densitometric analysis and were presented in the lower panel of each image. Values are presented as fold changes compared to the control group and are expressed as mean ± SEM, *n* = 3. In all of the experiments, * denotes *p* < 0.05 compared to the control cells; # denotes *p* < 0.05 compared with the cells treated with leptin alone.

Inflammasomes have been shown to be activated via multiple mechanisms. To clarify the signaling mechanisms underlying inflammasome activation by leptin, we investigated the potential role of ER stress. As shown in Figure 3A, leptin treatment induced significant increase in the levels of the chaperone protein GRP78/BiP in a time- (left panel) and dose-dependent (right panel) manner. Moreover, leptin markedly enhanced the expression of the genes that are related to ER stress; for example, it promoted the phosphorylation of PERK (Figure 3B) and eif2α (Figure 3C), up-regulated CHOP (Figure 3D), and increased the levels of active ATF6 (Figure 3E) in a time- and dose-dependent manner, clearly indicating that leptin induces ER stress in hepatocytes. In subsequent experiments for verifying the functional role of ER stress in inflammasome activation, pretreatment with tauroursodeoxycholic acid (TUDCA) significantly abrogated leptin-induced maturation of IL-1β (Figure 3F) and caspase-1 cleavage (Figure 3G) and downregulated the expression levels of NLRP3 (Figure 3H) and ASC (Figure 3I). In addition, as expected, decreased cell viability by leptin was restored by pretreatment with TUDCA (Figure 3J). TUDCA also suppressed leptin-induced caspase-3 activation (Figure 3K) and LDH release (Figure 3L), a pattern that is essentially similar to those observed upon treatment with inflammasome inhibitors. These results collectively suggest that ER stress plays a pivotal role in inflammasome activation and hepatocyte death induced by leptin. 

### 2.4. ROS Production Contributes to Leptin-Induced ER Stress and Inflammasome Activation in Rat Hepatocytes

**Figure 4:**
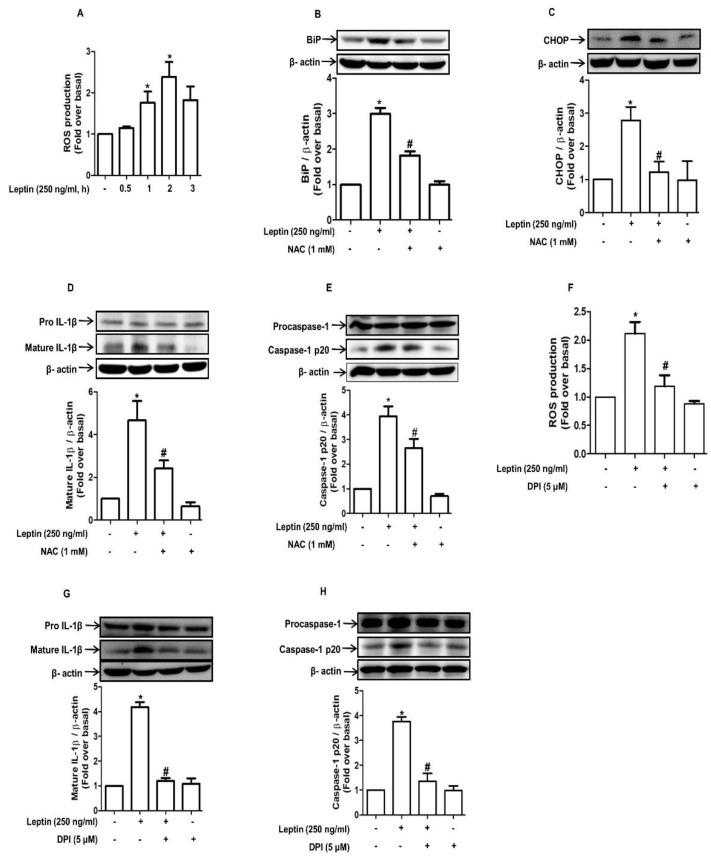
Role of ROS production in leptin-induced ER Stress and activation of inflammasomes in rat hepatocytes: (**A**) Cell were treated with leptin (250 ng/mL) for different time durations, and total ROS production was measured as indicated in the methods. (**B**–**E**) Hepatocytes were pretreaed with N-acetylcystein (NAC), a ROS scavenger, for 1 h followed by further treatment with leptin (250 ng/mL) for 8 h. Expression levels of BiP (**B**), CHOP (C), IL-1β (**D**), and caspase-1 (**E**) were measured by Western blot analysis. (**F**–**H**) Hepatocytes were pretreated with diphenyleneiodonium (DPI), a pharmacological inhibitor of NADPH oxidase, for 1 h and were further stimulated with leptin for an additional 8 h. (**F**) ROS production was assessed by measuring the fluorescence of DCF. (**G**,**H**) Expression levels of IL-1β (**G**) and caspase-1 (**H**) were determined by Western blot analysis. For Western blot analyses, expression levels of the genes were quantitated by densitometric analysis and are presented in the lower panel of each image. Values are presented as fold changes in comparison to the control group and are expressed as mean ± SEM, *n* = 3. * represents *p* < 0.05 compared to control cells; # represents *p* < 0.05 compared to the cells treated with leptin alone.

To further determine the upstream mechanisms that are involved in inflammasome activation, we investigated the role of ROS production. As shown in Figure 4A, leptin treatment significantly enhanced ROS production under our experimental conditions, which is consistent with previous reports. In addition, pretreatment with N-acetylcystein (NAC), a ROS scavenger, significantly suppressed leptin-induced BiP and CHOP expression (Figure 4B,C, respectively), indicating that ROS production is an upstream mechanism for ER stress induction. NAC also blocked leptin-induced IL-1β maturation and caspase-1 activation (Figure 4D and 4E, respectively), further confirming the role of ROS production in inflammasome activation by leptin. In the ensuing experiments that were conducted to identify the source for ROS production, we observed that leptin increased the expression of Nox1 and Nox2, which are subunits of the NADPH oxidase (Supplementary Figures S1 and S2). Moreover, treatment with diphenyleneiodonium (DPI), a pharmacological inhibitor of NADPH oxidase, almost completely inhibited leptin-induced ROS production (Figure 4F), IL-1β maturation (Figure 4G), and caspase-1 activation (Figure 4H), suggesting that the NADPH oxidase is a critical source for ROS production by leptin in hepatocytes.

### 2.5. Autophagy Induction Mediates Inflammasome Activation by Leptin in Rat Hepatocytes

**Figure 5:**
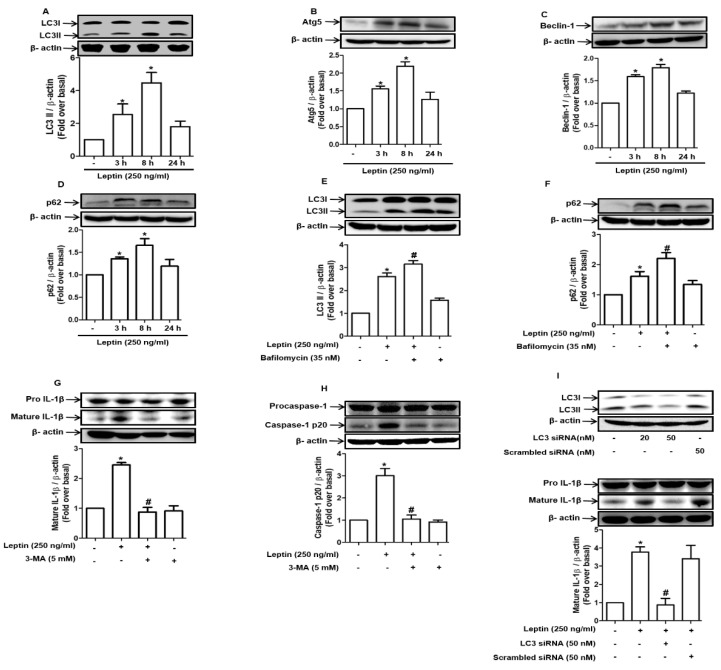

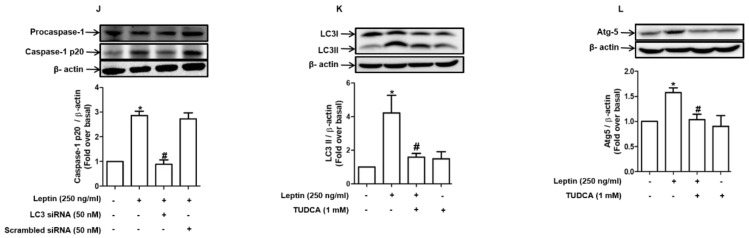
Role of autophagy induction in inflammasome activation by leptin in rat hepatocytes: (**A**–**D**) Hepatocytes were treated with leptin (250 ng/mL) for different time durations. Expression levels of the genes related to autophagy, including LC3I/II (**A**), Atg5 (**B**), Beclin-1 (**C**), and p62 (**D**), were measured by Western blot analysis. (**E**,**F**) Cells were pretreated with bafilomycin A, a lysosomal inhibitor, for 1 h followed by further stimulation with leptin for 8 h. Expression levels of LC3I/II (**E**) and p62 (**F**) were analyzed by Western blot analysis. (**G**,**H**) Hepatocytes were incubated for 1 h in the absence or presence of 3-MA followed by further incubation with leptin for 8 h. Active IL-1β (**G**) and caspase-1 (**H**) levels were determined by Western blot analysis. (**I**,**J**) Cells were transfected with the siRNA targeting LC3B or scramble control siRNA. After 24 h incubation, cells were further stimulated with leptin for 8 h. Active IL-1β (**I**) and caspase-1 (**J**) levels were assessed by Western blot analysis. Gene silencing efficiency of LC3B was monitored by Western blot analysis and is presented in the upper panel of (**I**). (**K**,**L**) Cells were pretreated with TUDCA for 1 h followed by treatment with leptin (250 ng/mL) for 8 h. Expression levels of LC3I/II (**K**) and Atg5 (**L**) were determined by Western blot analysis. For Western blot analyses, expression levels of the target genes were quantitiated by densitometric analysis and are shown in the lower panel of each image. Values are presented as fold change compared to the control group and are expressed as mean ± SEM, *n* = 3. * represents *p* < 0.05 compared to control cells; # denotes *p* < 0.05 compared with the cells treated with leptin alone or not transfected with siRNA.

Autophagy, which is commonly induced under stressful conditions, such as ROS production and ER stress, plays an important role in the modulation of cell death and survival. We further evaluated the role of autophagy induction in leptin-induced inflammasome activation. Leptin treatment induced the accumulation of LC3II (Figure 5A) and an increase in the expression of the genes related to autophagy, including Atg5 (Figure 5B), Beclin-1 (Figure 5C), and sequestome 1 (SQSTM1)/p62 (Figure 5D). To further characterize leptin-induced autophagy activation, we conducted an autophagy flux assay, which revealed that the expression levels of LC3II (Figure 5E) and p62 (Figure 5F) were further enhanced by treatment with bafilomycin A1, a lysosomal inhibitor, suggesting that the leptin-induced increase in the expression of autophagy-related genes may be attributed to autophagy induction rather than to the blockade of autophagic degradation. Moreover, 3-MA, an inhibitor of autophagy initiation, suppressed leptin-induced IL-1β maturation and the cleavage of caspase-1 (Figure 5G,H, respectively). The critical role of autophagy induction in inflammasome activation was further confirmed by the genetic ablation of LC3B. As shown in Figure 5, transfection with an LC3B-targeting siRNA also markedly abolished leptin-induced IL-1β maturation (Figure 5I) and caspase-1 activation (Figure 5J). Lastly, we further investigated the possible role of ER stress in leptin-induced autophagy activation. Pretreatment with TUDCA substantially blocked the leptin-induced increase in LC3II formation and Atg5 expression (Figure 5K,L, respectively), implying that ER stress contributes to inflammasome activation via autophagy induction in leptin-treated hepatocytes. 

### 2.6. Maturation of Cathepsin B Induced by Autophagy Promotes Inflammasome Activation in Rat Hepatocytes

**Figure 6:**
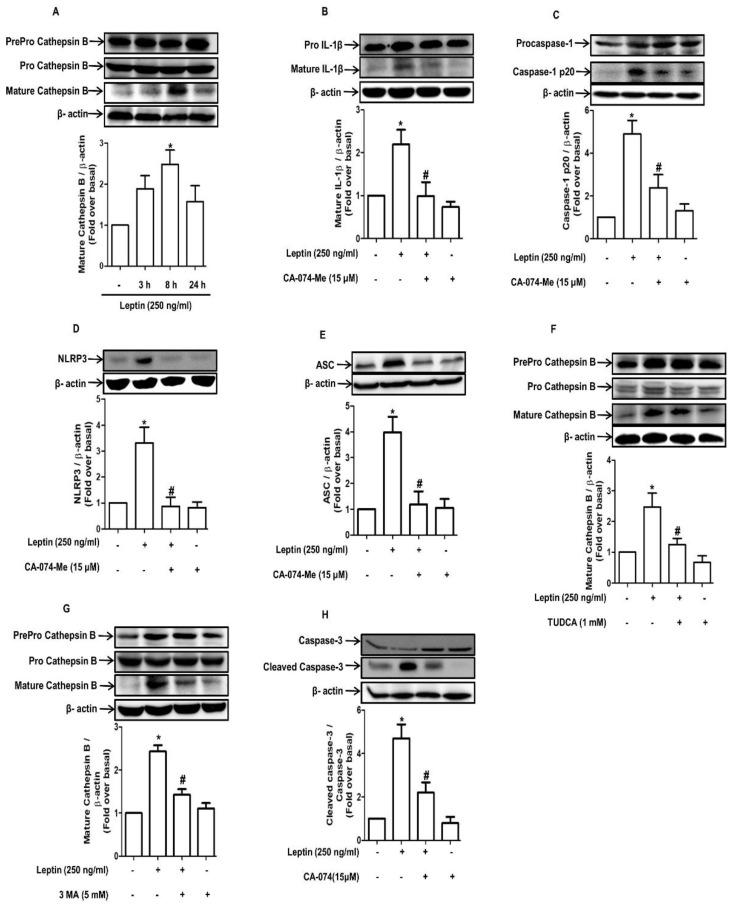
Effect of leptin on cathepsin B maturation and its involvement in inflammasome activation in rat hepatocytes: (**A**) Hepatocytes were treated with leptin for the indicated time durations. Expression of prepro-, pro, and mature cathepsin B were measured by Western blot analysis. (**B**–**E**) Cells were pretreated with CA-074-Me for 1 h followed by further incubation with leptin for 8 h. Expression levels of the genes related to inflammasomes, including pro- and mature IL-1β (**B**), pro- and active caspase-1 (**C**), NLRP3 (**D**), and ASC (**E**) were determined by Western blot analysis. (**F**,**G**) Rat hepatocytes were pretreated with TUDCA (**F**) or 3-MA (**G**) for 1 h and were further incubated with leptin for 8 h. Expression levels of prepro-, pro-, and mature cathepsin B were determined by Western blot analysis. (**H**) Cells were pretreated with CA-074-Me for 1 h followed by further stimulation with leptin (250 ng/mL) for 8 h. Cleaved active caspase-3 formation was assessed by Western blot analysis. For all the Western blot analyses, protein expression of the target genes was quantitated by densitometric analysis and is shown in the lower panel of each image. Values are presented with fold changes compared to the control and are expressed as mean ± SEM, *n* = 3. * represents *p* < 0.05 compared to control cells; # represents *p* < 0.05 compared to the cells treated with leptin alone.

Cathepsin B, a lysosomal protease, has been shown to induce inflammasome activation, and its activation process is mediated via ER stress in pancreatic cells [22]. We therefore investigated the potential role of cathepsin B in inflammasome activation by leptin. As shown in Figure 6A, leptin robustly increased the mature form of cathepsin B without exerting a significant effect on the prepro- and pro-form. Moreover, pretreatment with a cathepsin B inhibitor (CA-074-Me) significantly inhibited leptin-induced inflammasome activation, as evidenced by the suppression of IL-1β maturation (Figure 6B), caspase-1 cleavage (Figure 6C), and the downregulation of NLRP3 (Figure 6D) and ASC expression (Figure 6E), suggesting the potential role of cathepsin B in inflammasome activation by leptin. Leptin-induced maturation of cathepsin B was abrogated by pretreatment with TUDCA (Figure 6F) and 3-MA (Figure 6G), indicating that cathepsin B activation by leptin is mediated via ER stress and autophagy induction. Finally, the inhibition of cathepsin B by pretreatment with CA-074-Me suppressed leptin-induced caspase-3 activation (Figure 6H), confirming that cathepsin B mediates leptin-induced hepatocyte death via inflammasome activation.

### 2.7. Leptin Induces Inflammasome Activation and ER Stress in Rat Liver

**Figure 7:**
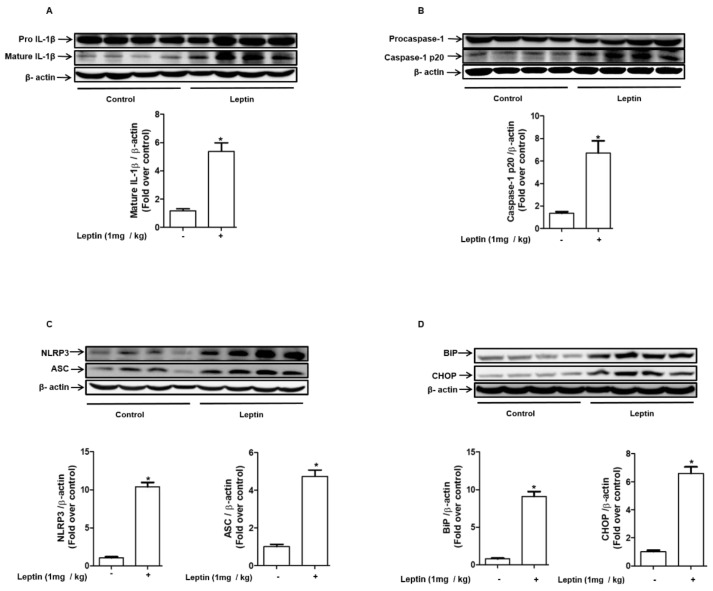
Effects of leptin on inflammasome activation and ER stress in rat liver: Rats were administered leptin for 10 days at a dose of 1mg/kg/day. After liver excision, total cellular extracts were prepared as described in the Methods section and were used for Western blot analysis to determine the expression levels of active IL-1β (**A**), caspase-1 (**B**), NLRP3, ASC (**C**), Bip, and CHOP (**D**), which are marker genes for inflammasomes and ER stress. Expression levels of the proteins were quantified by densitometric analysis and are presented in the lower panel of each image. Values presented as mean ± SEM, *n* = 4. * represents *p* < 0.05 compared to control rats.

To validate the in vitro effects of leptin on inflammasome activation under in vivo conditions, the expression levels of the maker genes for inflammasomses and ER stress were measured in rat liver after treatments with leptin. As shown in Figure 7, leptin administration induces significant increases in mature IL-1β (Figure 7A) and cleaved caspase-1 (Figure 7B). In addition, the expression levels of NLRP3 and ASC were substantially up-regulated (Figure 7C). Moreover, we also observed that leptin treatment induces the up-regulation of BiP and CHOP (Figure 7D). Taken together, these results verify the promoting effects of leptin on inflammasome activation and ER stress in rat liver in vivo. 

## 3. Discussion

Leptin has long received an attention as a master regulator of the physiological energy balance since it was first discovered as a hormone secreted by adipose tissue. Increasing recent evidence indicates that leptin signaling is also implicated in the development of various liver diseases, including steatosis, hepatitis, and fibrosis. Specifically, leptin upregulates collagen and smooth muscle-α-actin in hepatic stellate cells representing fibrotic changes [2]. In addition, it activates Kupffer cells, thereby generating inflammatory responses [23] and inducing lipid accumulation in hepatocytes [4]. Although leptin signaling is widely known to play important roles in various liver diseases by modulating different types of liver cells, there is limited knowledge regarding the cytotoxic effect of leptin in hepatocytes. In this study, we have examined whether leptin directly induces cytotoxicity in hepatocytes and have further elucidated the underlying mechanisms with a particular focus on inflammasomes. We have demonstrated that NLRP3 inflammasome activation critically contributes to the cytotoxic effects of leptin in hepatocytes. In addition, the activation of NLRP3 inflammasome activation by leptin is mediated, at least in part, via ROS production, ER stress, and autophagy-dependent mechanisms. To the best of our knowledge, this is the first report demonstrating the potential role of the ER stress/autophagy/inflammasomes axis in leptin-induced direct hepatocyte death and potential liver damage.

Inflammasome signaling is a platform for the activation of the immune system upon exposure to pathogenic stimuli and acts as a cell death mechanism in various liver diseases. For example, ER stress-mediated NLRP3 inflammasome activation and the hyperactivation of NLRP3 generated via genetic modification leads to pyroptotic death in hepatocytes [9,14], suggesting that inflammasome activation would be a potential mechanism for inflammation-mediated damage in liver cells. Moreover, besides the generation of inflammatory responses, active IL-1β signaling which is the final product of inflammasome activation, leads to apoptosis [24], necrosis [25], and pyroptosis [9]. In line with this notion, we observed that the inhibition of NLRP3 and caspase-1 protects hepatocytes from leptin-induced cytotoxicity (Figure 2F–H), and blockade of IL-1β signaling suppressed leptin-stimulated caspase-3 activation and LDH release (Figure 2I,J), clearly indicating that inflammasome activation plays a crucial role in mediating the cytotoxic effects of leptin in hepatocytes. As indicated earlier, inflammasome activation leads to various types of cell death by generating active IL-1β. Diverse mechanisms by which IL-1β signlaing induces cell death have been proposed, such as the modulation of the mitochondrial pathway of apoptosis [26] as well as the activation of p38MAPK [27] and JNK [28]. In the present study, we did not identify the detailed molecular mechanisms that link IL-1β signaling to leptin-induced apoptosis and pyroptosis. Further investigation of the mechanisms underlying cell death by IL-1β signaling would provide better insight into the hepatotoxic effects of leptin. Given the recent reports that other cytokines belonging to the interleukin family, including IL-18 and IL-33, also mediate biological responses by inflammasomes, it would be worth to investigate the involvement of other inflammatory cytokines. Notably, in contrast to these findings, recent evidence indicates that inflammasome activation leads to the suppression of apoptosis and the progression of cell cycle and therefore contributes to the survival of cancer cells and tumor growth [12,29,30], suggesting that the modulatory roles of inflammasomes in cell death and survival are controversial and depend on experimental conditions, including the stimuli used, duration of exposure, and cell type. Further studies investigating what determines the fate of the cells in response to inflammasome activation are needed to gain deeper insight into the inflammasome-mediated modulation of cell death and survival.

Prolonged ER stress causes various types of cellular dysfunction and eventually leads to cell death through coordinated regulation by multiple genes [31]. In this study, we clearly demonstrated that leptin induced an increase in the expression of genes that regulate ER stress in hepatocytes. Furthermore, enhanced ER stress was observed to crucially contribute to cell death via the inflammasome activation-dependent mechanism (Figure 3). Moreover, we also found that ER stress is a potential inducer of autophagy activation, which further mediates inflammasome activation (Figure 5). These findings collectively imply that ER stress–autophagy induction-inflammasome activation axis critically contributes to cytotoxic effects of leptin in hepatocytes. ER stress induces autophagy activation via multiple mechanisms, such as through the transcriptional up-regulation of the genes that are related to autophagy [32], the suppression of mTOR activity [33], and the dissociation of Bcl-2 and Beclin-1, which results in the generation of free Beclin-1 [34]. At this stage, we could not clearly understand the molecular interactions comprising ER stress, autophagy induction, and inflammasome activation. It has been hypothesized that ER stress transduces various signalings by Ca^2+^, ROS, and lipid-mediated pathways, leading to the overproduction of mitochondrial ROS, which promotes the activation of the NLRP3 inflammasomes [35]. Further exploration of the connection of ER and mitochondria on leptin treatment could provide better insight into the mechanisms for inflammasome activation by ER stress in hepatocytes upon leptin treatment.

Cathepsins, which are lysosomal cysteine proteases, have been shown to play an important role in protein recycling within the lysosome [36]. Recent studies have demonstrated that, in addition to the maintenance of protein homeostasis, cathepsin B activates the NLRP3 inflammasomes by directly interacting with NLRP3 through the LRR domain or by facilitating the interaction of the thioredoxin interacting protein (TXNIP) with NLRPs [37,38]. Moreover, cathepsin B-mediated inflammasome activation has been shown to modulate cell death and survival in various experimental models [39]. In this study, we observed that leptin significantly induced the maturation of cathepsin B (Figure 6A), and the inhibition of cathepsin B markedly prevented the leptin-induced activation of NLRP3 inflammasomes (Figure 6B–E). Moreover, autophagy inhibition by treatment with 3-MA prevented cathepsin B maturation (Figure 6G), collectively indicating that cathepsin B acts as a mediator for autophagy-induced inflammasome activation and that cathepsin B would be a novel pharmacological target for the treatment of the pathological conditions induced by leptin. To the best of our knowledge, this is the first report to provide evidence for the stimulatory effect of leptin on cathepsin B maturation and its involvement in the cytotoxic effects by leptin. Interestingly, it has been recently reported that autophagy and cathepsin B modulate their physiological activities in a complex manner. For instance, while we have shown the crucial role of autophagy induction in cathepsin B maturation, other studies have also revealed that the activation of cathepsin B conversely contributes to autophagy induction [40]. In this study, we also examined whether cathepsin B maturation is implicated in autophagy induction under our experimental conditions and observed that treatment with CA-074-Me abrogated leptin-induced Atg5 and LC3II accumulation (Supplementary Figures S3 and S4), suggesting that autophagy and cathepsin B may mutually regulate their activation processes, which further raises the possibility for a positive feedback loop in the modulation of their activity.

While a growing body of evidence suggests that autophagy is a process for cells to adapt to stressful conditions [41], autophagy is often accompanied with cell death after exposure to various insults [42]. Therefore, its role in the modulation of cell death and survival is controversial, and the underlying mechanisms determining the fate of the cells in response to autophagy remain elusive. In this study, we found that autophagy induction critically contributed to inflammasome activation by leptin in hepatocytes. Furthermore, co-treatment with 3-MA prevented leptin-induced caspase-3 activation (Supplementary Figure S5), confirming that the autophagic process is implicated in leptin-induced hepatocyte death. In contrast to these findings, a recent report showed that autophagy induction can protect hepatocytes from acetaminophen and myocardial ischaemia-reperfusion injury-induced cell death by modulating inflammasome activation [7,43], indicating the controversial role of autophagy in the regulation of inflammasome and cell death. The dual role of autophagy in the modulation of cell death and survival has been proposed to depend on the rate of autophagic flux, the duration of activation, and the ultimate fate of the recycled components in the autolysosomes [44]. We could not thoroughly address the mechanisms that are involved in the opposite roles of autophagy in the modulation of cell death in this study. Further studies are required to figure out the process by which autophagy differentially determines the fate of the cells.

## 4. Materials and Methods

### 4.1. Materials

Recombinant mouse leptin (L3772), N-acetyl cysteine (A9165), Ac-YVAD-CMK (SML0429), DPI (CN-240-0010), 3-methyladenine (M9281), type IV collagenase (C5138), type I collagen (C3867), and CA-074-Me (205530) were purchased from Sigma-Aldrich (St. Louis, MO, USA). Bafilomycin A1 (1829-50) and tauroursodeoxycholic acid (TUDCA, HY-19696A) were obtained from Biovision (Milipitas, CA, USA) and Medchem Express (New Jersey, USA), respectively. The primary antibodies against BiP (3183), ATF6 (65880), total PERK (3192), total eif2α (9722s), phospho-eif2α (9721s), beclin-1 (3738), LC3 (2775), p62 (5114), cleaved caspase-3 (9664), pro-caspase-3 (9662), Gasdermin D (39754), and IL-1β (12507) were purchased from Cell Signaling Technologies (Danvers, MA, USA); caspase-1 (AG-20B-0042) and ASC (AG-25B-0006) were procured from Adipogen (San Diego, CA, USA); Phospho-PERK (MA5-15033) and ATG5 (PA1-46178) were purchased from Thermo Fisher (Rockford, IL, USA); NOX1 (NBP1-31546), NLRP3 (MAB7578), and Cathepsin B (ab58802) were obtained from Novusbio (Littleton, CO, USA), R&D systems (Minneapolis, MN, USA), and Abcam (Cambridge, UK), respectively. 

### 4.2. Isolation and Culture of Hepatocytes

All the animal experiments were conducted in accordance with the guidelines of the Yeungnam University Institutional Animal Care and Use Committee (IACUC) (Approval number: LAC 2018-084). Hepatocytes were isolated from male Sprague Dawley rats (6–7 weeks old) via a two-step collagenase perfusion process, as described previously [45]. Briefly, the hepatic portal vein was cannulated using an 18G catheter, and the liver was perfused with HBSS (free of Ca^2+^ and Mg^2+^) followed by type IV Collagenase (0.05%). After the digestion of the liver tissues, the cells were harvested by centrifugation at 50× *g* for 30 s, washed with HBSS, and seeded in collagen-coated dishes. Cell viability was determinied by trypan blue exclusion assay. Cells were used for further experiments when the viability was more than 90%.

### 4.3. Measurement of Cell Viability

Cell viability was measured by MTS assay, as described previously [46]. Briefly, cells were seeded in collagen-coated 96-well plates (clear) at a density of 5 × 10^4^ cells/well. After overnight culture, the cells were incubated with leptin for 48 h and were further treated with MTS [(3-(4,5-Dimethylthiazol-2-yl)-5-(3-carboxymethoxyphenyl)-2-(4-sulfophenyl)-2H-trtrazolium) solution (20 μL) for 2 h at 37 °C. Cell viability was determined based on the conversion of MTS salt into a formazan product by measuring the absorbance at 490 nm using a SPECTROstar Nano microplate reader (BMG LABTECH, Allmendgrün, Ortenberg, Germany).

### 4.4. Preparation of Cellular Extracts and Western Blot Analysis

Hepatocytes were plated in collagen-coated 35 mm dishes at a density of 1 × 10^6^ cells. The control cells were seeded for the same duration as leptin treated cells. After treatments with leptin and the respective inhibitors, cells were extracted with RIPA lysis buffer containing a protease inhibitor cocktail. For in vivo study, Sprague Dawley rats (6–7 weeks old) were administered with leptin for 10 days at a dose of 1mg/kg/day via intraperitoneal injection. The control rats received PBS at an equivalent volume. The animals were maintained in standard housing conditions and were fed with a standard diet and water ad libitum with 12 h light/dark cycle. After treatments, the animals were sacrificed, and the liver was then excised and stored at −80 °C for subsequent experiments. The tissues were homogenized in RIPA buffer containing 1% protease inhibitors and were further lysed using a sonicator. 

For Western blot analysis, total proteins (30–40 μg) were loaded in a sodium dodecyl sulfate polyacrylamide gel (8–15%), separated by electrophoresis, and transferred to PVDF membranes. The membranes were blocked with non-fat dry skim milk for 1 h, incubated with the designated primary antibody (in PBS-Tween supplemented with 3% BSA) overnight at 4 °C, and further incubated with the secondary antibody conjugated with horseradish peroxidase for 1 h. All primary antibodies, except β-actin, were used at a 1:1000 dilution. β-actin was diluted at a ratio of 1:5000, and the secondary antibodies were diluted at a ratio of 1:4000. Chemiluminescent images were captured using a Fujifilm LAS-4000 mini (Fujifilm, Greenwood, South Carolina, USA).

### 4.5. Measurement of Reactive Oxygen Species (ROS) Production

Total ROS production was measured as previously described [47]. Briefly, hepatocytes were seeded at a density of 5 × 10^4^ cells/well in a collagen coated 96-well black plate. After treatments, hepatocytes were incubated with 5-chloromethyl-2′,7′-dichlorodihydrofluorescein diacetate (CM-H2DCFDA, 5 μM) for 30 min in the dark. ROS production was quantified by the change in the fluorescence intensity of CM-H2DCFDA using a FLUO star OPTIMA fluorimeter (BMG LABTECH, Inc, Allmendgrün, Ortenberg, Germany).

### 4.6. Measurement of Lactate Dehydrogenase (LDH) Release

LDH release was determined using the CytoTox 96 Non-Radioactive Cytotoxicity Assay Kit (Promega Corporation, Madison, WI, USA) according to the manufacturer’s instructions. Briefly, hepatocytes were seeded at a density of 5 × 10^4^ cells/well in collagen-coated clear 96-well plates. After treatment with leptin, the cell culture media (150 μL) were collected and incubated with LDH reagent (20 μL) for 1 h in the dark. The quantity of LDH that was released was measured based on the conversion of a tetrazolium salt into a formazan product by measuring the absorbance at 490 nm using a Spectrostar Nano microplate reader (BMG LABTECH).

### 4.7. Immunocytochemistry for the Detection of ASC Speck

ASC speck formation was detected as previously described [7]. Briefly, hepatocytes were seeded at a density of 5 × 10^4^ cells/well in collagen-coated 8-well chamber slides. After treatment with leptin, cells were fixed with 4% paraformaldehyde, permeabilized with 0.1% Triton X-100, and blocked using 1% BSA in PBS for 1 h. Cells were then incubated overnight with a primary antibody against ASC (1:300) at 4 °C and with FITC-conjugated goat anti-rabbit secondary antibody for 1.5 h in dark, and they were further incubated with DAPI. Fluorescent images of the ASC specks were captured by an A1 confocal laser microscope system (Nikon Corporation, Tokyo, Japan).

### 4.8. Transient Transfection with Small Interfering RNA (siRNA)

Rat hepatocytes were seeded in a collagen-coated 35–mm dish at a density of 5 × 10^5^ cells. After overnight culture, cells were transfected with siRNA targeting LC3B or scrambled control siRNA for 24 h using Lipofectamine RNAi Max (Thermo Fisher, Rockford, IL, USA) according to the manufacturer’s instructions. The gene silencing efficacy was monitored by Western blot analysis after 24 h of transfection. Oligonucleotide siRNAs targeting LC3B gene were purchased from Bioneer (Daejon, Korea). The sequences of the siRNAs were as follows:

Forward: 5′-GUCACUCACUCGUGUCUGA-3′;

Reverse: 5′-UCAGACACGAGUGAGUGAC-3′.

### 4.9. Statistical Analysis

Values are presented as mean ± S.E.M. from at least three independent experiments. Data were analyzed by One-Way Analysis of Variance and Tukey’s multiple comparisons test and t-test using GraphPad Prism software version 5.0. Differences between groups were considered to be significant at *p* < 0.05.

## 5. Conclusions

**Figure 8:**
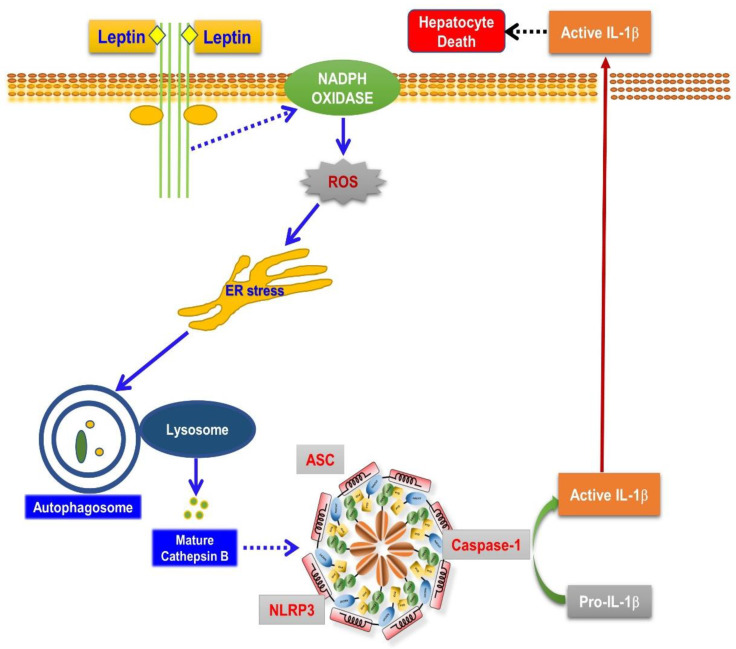
Proposed model for inflammasome activation by leptin and its role in hepatocyte death: Leptin induces apoptotic and pyroptotic cell death in rat hepatocytes. Cytotoxic effect of leptin in hepatocyte is mediated via activation of NLRP3 inflammasomes, which results in cleavage of caspase-1 and the maturation of IL-1β. NLRP3 inflammasome activation by leptin is initially mediated via ROS production derived from NADPH oxidase. Enhanced intracellular ROS levels causes prolonged ER stress, which leads to autophagy induction and the further maturation of cathepsin B. Moreover, cathepsin B activation critically contributes to NLRP3 inflammasome activation, indicating that the ROS production/ER stress/autophagy induction/cathepsin B maturation axis is implicated in inflammasome activation by leptin in hepatocytes. Detailed molecular mechanisms by which active cathepsin B mediates inflammasome activation and IL-1β signaling leading to apoptotic and pyroptotic cell death in hepatocytes remain to be further investigated.

The present study has demonstrated that leptin induces apoptotic and pyroptotic cell death in hepatocytes via the activation of NLRP3 inflammasomes, which is mediated, at least in part, via the ROS/ER stress/autophagy/cathepsin B axis (Figure 8). To the best of our knowledge, this is the first report to demonstrate the critical role of inflammasome activation in leptin-induced direct hepatocyte death and potential liver damage. Modulation of inflammasome activation would be a potential therapeutic strategy for the protection of hepatocytes from leptin-induced cytotoxicity.

## Data Availability

The data presented in this study are available upon request from the corresponding author.

## Supplementary Materials

The following are available online at https://www.mdpi.com/article/10.3390/ijms222212589/s1. Figures S1 and S2: Effects of leptin on Nox-1 and Nox-2 expression in rat hepatocytes. Figures S3 and S4: Role of cathepsin B in autophagy induction by leptin in rat hepatocytes. Figure S5: Role of autophagy induction in leptin-induced caspase-3 activation in rat hepatocytes.
