# Supplementary figures and images for: Leptin Induces Apoptotic and Pyroptotic Cell Death via NLRP3 Inflammasome Activation in Rat Hepatocytes
Source: Int J Mol Sci. 2021 Nov 22;22(22):12589. doi: 10.3390/ijms222212589 (PMC8622994; doi:10.3390/ijms222212589)

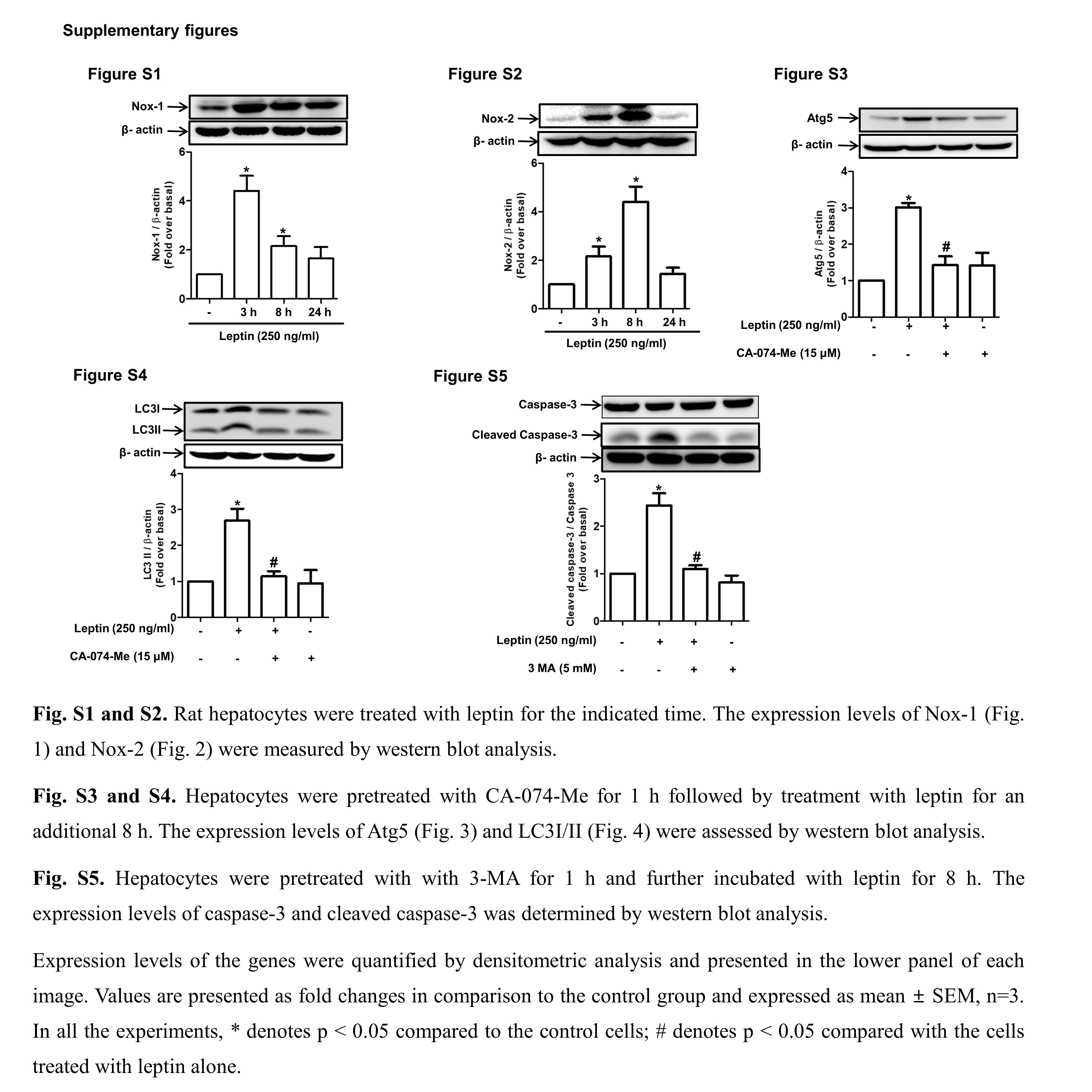

Supplement: Supplementary file 1 [file ijms-22-12589-s001.zip › ijms-1453854-supplementary.jpg]
